# 3D Master Toothguide Is Adequate to Subjective Shade Selection?

**DOI:** 10.3390/medicina58030457

**Published:** 2022-03-21

**Authors:** Cristina Gómez-Polo, Miguel Gómez-Polo, Norberto Quispe López, Maria Portillo Muñoz, Javier Montero

**Affiliations:** 1 Department of Surgery, Faculty of Medicine, University of Salamanca, 37007 Salamanca, Spain; norberto_quispe@usal.es (N.Q.L.); mportillo@usal.es (M.P.M.); javimont@usal.es (J.M.); 2 Department of Orofacial Prosthesis, Faculty of Dentistry, Complutense University of Madrid, 28001 Madrid, Spain; mgpolo@odon.ucm.es

**Keywords:** spectrophotometry, natural tooth colour, 3D Master System, dental shade guide, intermediate shades, CIELAB

## Abstract

*Background and Objectives:* To study the validity and the degree of representability of the toothguide 3D Master, with 26 physically shade tabs, on the natural tooth colour on a sample of the Spanish population. *Materials and Methods:* Natural tooth colour was measured in a sample of 1361 Spanish participants of both genders distributed within an age range of 18 and 89 years of age. The colour coordinates were calculated and the frequency of the 26 physically shade tabs of the toothguide as well as the “intermediate shades” (without physical representation in toothguide) through the Easyshade Compact (Vita-Zahnfabrik) spectrophotometer using the 3D Master System nomenclature. The colour differences between the “intermediate shades” were calculated using the Euclidean formula (ΔEab*). The program used for the present descriptive statistical analysis of the results was SAS 9.1.3. *Results:* A total of 49 “intermediate shades” were registered in 816 participants (60%). The colour coordinates of the 49 ‘intermediate shades’ cover colour coordinates ranging from 0M1.5 (L* 100.0, C* 7.70, h* 112.2) to 5M2.5 (L* 56.8, C* 35.8, h* 78.5). Not all possible 3D Master System’s “intermediate shades” were registered in the population studied. 82.4% of the colour differences among the “intermediate shades” were clinically unacceptable (ΔEab* ≥ 5.5 units). *Conclusions:* Only 40% of the population studied presented a natural tooth colour belonging to the 3D Master Toothguide’s physical shade tabs.

## 1. Introduction

The 3D Master colour system (VITA Zahnfabrik, Bad Säckingen, Germany), 26 physical shades tabs and "intermediate shades" (without physical representation), was the most complete and versatile commercially available system for measuring tooth colour to date [1,2,3,4,5,6,7]. According to its manufacturer, it offered the possibility of determining, in a safe, scientific, and systematic way, all natural tooth shades, according to the three-dimensions of colour: brightness, chrome, and hue. According to the 3D Master system, tooth colour should have been selected in the following order: first value or lightness, then chrome, and finally hue [8]. Consequently, each colour was determined by a number (value or lightness group), a letter (hue group), and a number (chrome group), such as 2L1.5, where 2 represents lightness group, L represents hue group and 1.5 represents chrome group. Letter L showed a higher quantity of yellow, letter R, a larger quantity of red, and letter M indicated an intermediate hue [9].

The subjective selection of colour, through a direct comparison between the physical shade tabs from the tooth guides and the vestibular surface of the tooth, was the most widely used technique in dentistry [10]. The objective technique, using electronic devices, offered the possibility of providing the result according to the tooth guides classification, and/or in objective colour coordinates, eliminating the subjective choice of the operator [7,11,12,13,14].

In 1976, the International Commission on Illumination defined colour spectrum through the ordinate coordinates on the three space axes: L* measurement of an object’s value (L* = 0 black; L* = 100 white); a* red-green measurement axis (a* positive indicates the amount of red and a* negative indicates the amount of green); and b* yellow-blue measurement axis (b* positive indicates the amount of yellow and b* negative indicates the amount of blue). The CIE 1976 L* a* b* identifies the components of the cylindrical coordinates CIE L*, C*, h*: lightness, chrome, and hue, respectively. The circular axis was known as h*. The units were in the form of degrees (or angles), ranging from 0° (red) through 90° (yellow), 180° (green), and 270° (blue) [15]. The L* parameter did not change, since it corresponded to the vertical axis. To quantify the difference between two colours, the most widespread dental formula was derived from the CIE-L*a*b* system, ∆Eab* = [(∆L*) ^2^ + (∆a*)^2^ + (∆b*)^2^ ]^1/2^. ∆Eab* represented the magnitude of the colour difference [16,17,18].

No standardised value of ΔEab* provided a threshold for acceptability or perception in dental colour matching. In 2013, Khashayar et al. published an article that aimed to establish, through available literature, what is currently considered the perceptibility threshold and acceptability threshold for colour difference value in dental research [19]. One of the few in vivo studies was the one published in 2008 by Douglas et al. [20], which was commonly referred to in experiments related to colour differences. In this study the mean colour perceptibility tolerance for perceptibility for 50% of observers was 2.6 ΔEab*units. The mean acceptability threshold was 5.6 ΔEab* units, which were the reference values we used in this study [20]. The basic principle on which the 3D Master colour system was based, and where its advantages lay, was that it covered the spectrum of colours which made natural tooth colour, in a homogeneous, orderly and uniform manner, according to the three dimensions of colour. Therefore, the “intermediate shades”, which lacked physical representation, were obtained by mixing the closest 26 basic colours in a 50% proportion. The 3D Master Toothguide had been widely studied [20,21,22], unlike the “intermediate shades”. These “intermediate shades” could only be obtained through spectrophotographic measurements, since there was no physical representation of them in the 3D Master toothguide [22]. In order to ensure that the “intermediate shades” complied with the manufacturer’s guidelines for their chromatic obtainment, as well as to ensure quality control over the “intermediate shades” of prosthetic restorations, we needed to know the objective colour coordinates. These disadvantages could have been minimised if a reliable basis for colour coordinates would have been obtained for each shade of the 3D Master System (26 physical shade tabs and “intermediate shades”) [23]. The arbitrariness which governs the reasonable resemblance between the colour of prosthetic dental restorations and the natural colour of the adjacent tooth may have been diminished if we would have standardised the colour coordinates for each colour of the 3D Master system.

Many researchers have studied natural tooth colour [5,24,25,26,27,28,29], but these studies presented their results only through the colour coordinates according to the CIELAB System and not through the 3D Master System nomenclature. The objectives of this study were (1) to study if the 3D Master toothguide is valid and representative of the natural tooth colour on a sample of the Spanish population, (2) to know the colour coordinates of the “intermediate shades”, and (3) to study colour differences between “intermediate shades”.

The null hypothesis relied on the fact that the 26 physical shades tabs of 3D Master Toothguide were representative and adequate to determine the natural tooth colour on a sample of Spanish population.

## 2. Materials and Methods

**Figure 1:**
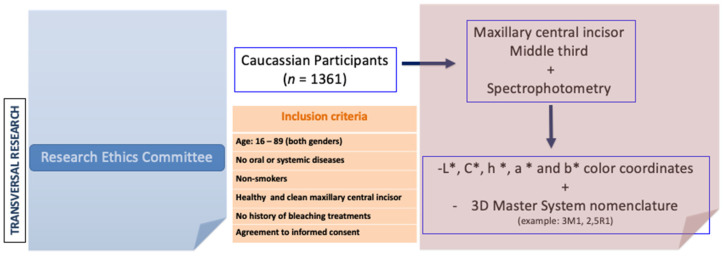
Graphical representation of methodology.

Measurements were obtained from a sample of 1361 maxillary central incisors belonging to 1361 homogeneously distributed in Caucasian men (*n* = 671) and women (*n* = 690) aged between 18 and 89. The inclusion criteria for participants in this study were: no oral or systemic diseases, non-smokers, healthy maxillary central incisor, and no history of bleaching treatments (Figure 1). The sample was divided, according to age, into eight decades. The chi square test for homogeneity, with *p* > 0.05, revealed that there were no statistically significant differences among groups regarding age and gender (X^2^ = 4550; 7 gl; *n* = 1361; *p* = 0.715). All participants were asked to read and sign an informed consent form in order to participate in the following study. The Research Ethics Committee of the San Carlos Clinic (Madrid) gave a positive evaluation of the protocol used in this study; in other words, it met the standards of good clinical practice. Thus, the clinician collected data from different places and a large part of the Spanish territory was covered: Caceres; Salamanca; Oviedo; Vigo; Toledo; Badajoz; Valladolid; Burgos; Leon; Soria; Zamora; Palencia; Ciudad Real; Avila; Madrid; and Zaragoza. All participants were evaluated at the Department of Prosthodontics, School of Odontology, Complutense University of Madrid, Spain.

The natural maxillary central incisors were healthy, unrestored, and unbleached, and they were cleaned before undergoing tooth colour measurement through spectrophotometry. In addition, the background colour was neutralised with grey gauze. All colour recordings were carried out under daylight lamps TLD95/65 with a luminous chrome of 1500 lux. One clinician (a woman of 30 with 8 years’ experience) participated in the study, always using the same Easyshade compact (VITA Zahnfabrik, Bad Säckingen, Germany). The examiner was instructed theoretically and practically in the handling of Easyshade Compact over two days. The spectrophotometer Easyshade Compact was a digital wireless device with a light source terminal, a screen and a probe tip to measure tooth colour in the diameter where it is placed. The Easyshade Compact (VITA Zahnfabrik, Bad Säckingen, Germany) enabled reliable digital measurement of tooth shades within a few seconds through its probe tip of 5 mm in diameter, composed of 19 optic fibres. One spectrophotometer used was the Vita Easyshade Compact (VITA Zahnfabrik, Bad Säckingen, Germany) (reg. no. H20394), which previously underwent a reliability test to check its temporal stability and inter-rater reliability. Colour was determined in the middle third of the tooth, which is the area that best illustrated tooth colour. Measurements of the tooth were made in “Single Tooth” mode. The target tooth was measured by holding the probe tip at 90 degrees to the surface in the middle third of the tooth.

**Figure 2:**
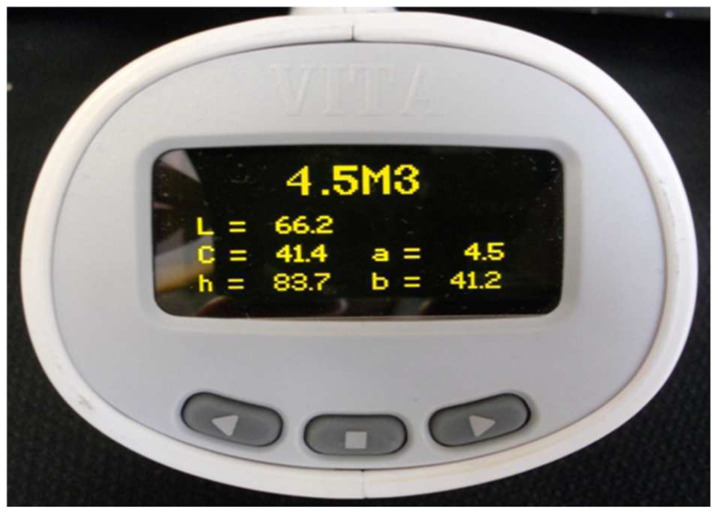
The Easyshade compact spectrophotometer.

The Easyshade compact was calibrated by placing a probe tip on the calibration port aperture before measuring each tooth. According to the manufacturer’s instructions, the measurement was accepted when two consecutive, identical readings were generated for each tooth. The objective method: the first result that matched twice was recorded according to the CIELab space coordinates L* (Lightness), C* (Chroma), and h* (hue), and according to the 3D Master System: 26 physical shade tabs, for example, 2M3, 4L2.5, or “intermediate shade”, for example 4.5M2.5, 3R2 (Figure 2). The Easyshade compact spectrophotometer recorded tooth colour coordinates according to the 3D Master System (26 physical shade tabs and intermediate shade tabs in the same way). In this text the mean orthogonal colour coordinates (CIELAB System), as well as the polar colour coordinates (CIELCh System) of the “intermediate colours” of 3D Master system, were presented. In addition, Euclidian equation ΔEab* ([(∆L*)^2^ + (∆a*)^2^ + (∆b*)^2^ ]^1/2^ was used to calculate the colour difference between the 49 "intermediate shades" of the 3D Master System. The program used for the descriptive statistical analysis of the results was SAS 9.1.3 statistical package software (100 SAS Campus Drive Cary, NC 27513-2414, USA).

## 3. Results

The natural tooth colour of 1361 participants was measured, of which 816 (60%) presented a colour without physical representation in one of the 26 shade tabs of the 3D Master Toothguide. Furthermore, only two subjects presented an “intermediate shade” with a group of lighthtness 0, a group unique to whitened teeth, according to the manufacturer.

Table 1 summarises the mean colour coordinates of both the CIELAB and the CIELCH system. It also indicates the colour frequency and percentage of each “intermediate shade”. Within the population studied 49 “intermediate shades” were recorded. Not all colours of the 3D Master System were represented, for example, or 2L1, 3L1, and 4.5M2.5.

The sample size of each of the “intermediate shades” was not similar. In fact, there were “intermediate shades” with a single representative as 0M1.5, 0.5M2, 2M1.5, and 2M2.5. On the other hand, the “intermediate shades” were most often 1M1.5 with 94 subjects, followed by 2.5L1.5 with 68 participants. The colour coordinates of the 49 “intermediate shades” covered colour coordinates from 0M1.5 (L* 100.0, C* 7.70 *, h* 112.2) to 5M2.5 (L* 56.8, C* 35.8, h* 78.5).

The L* coordinate was related to the lightness group (first number of the “intermediate shades”). It could be observed in Table 1 that as the lightness group of the “intermediate shades” increased, the L* coordinate gradually decreased. The C* coordinate was related to the chroma, and it could be observed that the colour intensity increased, reaching its peak in the “intermediate shade” 5M2.5, but that coordinate was not maintained in the same chroma groups. In other words, not all groups of chroma 2.5 or chroma 1 had similar C* coordinates. For example, in groups of chroma 2: 0.5M2 presented a C* coordinate of 95.9 units, 3R2 had a C* coordinate of 83.5 and 4L2 a C* coordinate of 88.6. With respect to the h* coordinate, one would expect that the L dyes would have had approximate scores of 90° and that the R dyes would have been around 0°, yet it was seen that this was not the case.

In Table 2, a colour difference (ΔEab*) of between 2.6 and 5.5 units was found in 177 pairs out of the 1176 pairs compared (15.0%). This was regarded as a detectable, but clinically acceptable difference. In 969 pairs (89.54%) there was a clinically unacceptable colour difference, exceeding the threshold of 5.5 units. Only 2.6% of the colour differences (ΔEab*) were less than 2.6 units.

## 4. Discussion

Derived from the data collection system, one can observe that not all colours have had the same frequency, whereupon the average coordinates may have been affected. Moreover, in several colours they were representative only. Not all possible “intermediate shades” of the 3D Master System were represented in the sample population used. It would have been interesting to replicate the study in other populations to see if the “intermediate shades” without representatives in this research would remain consistent or change. The utility in the precision of the subjective shade determination was questioned through the 26 physically shade tabs of toothguide 3D Master. The null hypothesis must be rejected because the 26 physical shade tabs are not enough to represent the colour of the population studied; 60% of the participants had a colour belonging to the “intermediate shades”. This implies the necessity of knowing the “intermediate shades” in order to offer a valid subjective shade determination in our patents.

Many publications on this issue have used a sample similar in size, or even smaller, than that of the present study, to draw conclusions for different populations [5,24,25,26,27,28,29]. All of these studies had in common the use of electronic devices in order to quantify the colour coordinates, but they did not all use identical instruments; a spectrophotometer, was used [5,25,27,28], as were spectroradiometric devices [26] and colorimeter devices [29]. The present investigation used the Easyshade compact spectrophotometer which had the advantage of being able to offer the results through the colour coordinates as well as in the 3D Master System nomenclature, which made it possible to correlate both types of measure for the tooth colour. In this way it was possible to obtain the colour coordinates of the “intermediate shades”. On the other hand, the maxillary central incisor has been frequently used to assess tooth colour, as representative of the natural tooth colour of a person [30,31] and the middle area was the most representative of a tooth’s colour as well [27,28,32,33]. This is why it was used in the present work. The position of the probe tip of the spectrophotometer on the tooth surface is important when obtaining colour coordinates. A standardised tooth positioner was not fabricated because the total number of the teeth was too great (1361 teeth) and their shape was too varied. Since no positioners are used for each participant, it is not possible to guarantee that the probe tip will always be repositioned in exactly the same place. Spectrophotometers are designed for smooth surfaces, an essential condition for reliable measurements to be obtained. In this sense, their use in dentistry is hindered by the convex surface of teeth, which complicates the correct placement of the spectrophotometer’s probe tip and captures roughly 25% of the colour reflected back from the tooth surface measured [25]. This study is not limited to the 26 physical shade tabs of the Toothguide 3D Master. We use a large population with a range of ages to obtain a sufficient sample for “intermediate shades”. This study is limited to the Caucasian population and cannot be extrapolated to other races.

Despite this, this study represented an approximation which could be used to carry out quality control of the colour composition of the “intermediate shades”. The lack of physical representation of the “intermediate shades” made rigorously obtaining the “colour ingredients” of each colour more complicated. In order to obtain them, one had to reply on the manufacturer’s guidelines. The manufacturer said that one must blend the closest of the 26 physical colours in order to obtain the intermediate colours. For example, to have gotten the colour 2.5M1.5, one must have mixed in a 50:50 ratio the 3D Master Toothguide colours 2M1 and 3M2. This assumed an evenly spaced spatial distribution of colour. Therefore, all the results obtained were objective and have been exclusively collected via spectrophotometer. The colour coordinates of the “intermediate shade” were essential in order to reproduce such colours accurately and safely and it could have been helpful in controlling the accuracy of colour resulting from mixtures of 50% of the 26 physical shade tabs of the 3D Master Toothguide of prosthetic dental restorations. It was necessary to take a dental shade match with a spectrophotometer in order to achieve precision in the natural tooth colour sample and avoid colour differences with negative clinical impact. That is, the colour of more than 50% of the population is not found among the 26 physical shade tabs of the Toothguide 3D Master. This means that it is important to take natural tooth colour through electronic devices, because it is impossible to choose “intermediate shade tabs colours” using subjective visual comparative. A colour matching system is more likely to be accurate in terms of tooth colour selection if a wide range of chromatic variation is available. “Intermediate shades” can only be measured by using a spectrophotometer. The accurate reproduction of “intermediate shades” in prosthetic restoration is problematic if the colour coordinates L*, C*, h*, a*, and b* are not distributed equally, according to the manufacturer. With this knowledge we can then confidently approach the 3D Master’s colour system comprising more than 75 dental colours.

As could be observed in Table 2, almost 90% of the colour differences between the “intermediate shades” carried a clinically unacceptable error. The present results of the colour differences (ΔEab*) among the “intermediates shades” of the 3D Master system were similar to the colour differences among the 26 physically shade tabs of the 3D Master toothguide [34]. This supported the idea that approximately 80% of the colour differences among the colours of the 3D Master system would exceed the clinical acceptance threshold of the patent (ΔEab* ≥ 5.5 units) [20]. Based on these results it seemed convenient to reconsider the design of the physical tabs of the colour guides with the purpose of being more precise and rigorous in the subjective dental colour selection in order to cover the entire dental colour spectrum. This would have implied a negative assessment of the restoration by the patient.

Thus, to study the dental colour coordinates through objective methods of the different population was basic in order to create representative colour tabs of the natural dental dentition. It was necessary to take a colour sample with a spectrophotometer in order to achieve precision in the natural tooth colour sample and avoid colour differences with negative clinical impact.

To date there have been no other related investigations that have provided the median colour coordinates of the Master 3D System’s “intermediate shades”, which complicates direct comparison of the present results.

These results offered an approximation of the 3D Master system’s “intermediate shades”, since the complete system was not represented and one did not have a homogeneous sample size of all the colours, which limited the results of the mean coordinates. It would have been very useful to create all possible mixtures of the porcelains of 50% of the 26 physical colours of the 3D Master Toothguide in order to obtain porcelain samples of the “intermediate shades” and to carry out a comparison with the present results. The 3D Master Toothguide offered the advantage of being able to measure on the surface of the 26 physical shade tabs, thus allowing one to obtain the colour coordinates. It could be suspected that the multitude of studies on the 3D Master Toothguide [8,9,10] and the lack of studies on the “intermediate shades” [6] may have been due to this phenomenon.

The colour coordinates of the “intermediate shade” were essential in order to reproduce such colours accurately and safely. Knowledge of the coordinates of the “intermediate shades” could have been helpful in controlling the accuracy of colour resulting from mixtures of 50% of the 26 physical shade tabs of the 3D Master Toothguide of prosthetic dental restorations. The “intermediate shades” did not mathematically meet the claims of the manufacturer, since the colour coordinates were not ordered mathematically and equidistantly on the colour spectrum, according to its three dimensions. The coordinate which appeared most faithful to the initial planned design was the coordinate L*, related to the brightness. This result was consistent with the results published by Gómez Polo et al. [10,11,18], in the study of the 3D Master Toothguide. The lack of knowledge and the lack of studies of the complete 3D Master System greatly limited its correct use and the wide chromatic possibilities offered by the system launched by Vita Zahnfabrik in 1998. In order to achieve an exact shade determination, the subjective method of the 3D Master toothguide might not be valid nor representative of the dental colour of the studied population. Moreover, the necessity of designing a colour guide with physical tabs representing the natural dental colour was noted.

## 5. Conclusions

In the population studied:

- Only 40% presented a natural tooth colour belonging to the 3D Master Toothguide’s physical shade tabs;
- The colour coordinates of the 49 intermediate shades are not mathematically equally distributed in CIELAB colour space;
- The 89.5% of the colour differences between 49 intermediate shades of the 3D Master System were a clinically unacceptable colour difference (ΔEab* > 5.5 units).

## Figures and Tables

**Table 1:** Colour coordinates of the 49 “Intermediate shades” registered in the 3D Master System.

Intermediate Shades of 3D Master System (Mean ±SD)						
Shades	*n*	L*	C*	h*	a*	b*
**0M1.5**	1	100.0	7.7	112.2	−1.4	7.1
**0.5M2**	1	92.3	13.7	95.9	−1.4	13.6
**1M1.5**	94	85.4 ± 2.0	16.2 ± 1.1	94.8 ± 1.8	−1.3 ± 0.5	16.2 ± 1.1
**1.5M1**	14	85.7 ± 3.3	11.6 ± 2.55	98.6 ± 2.7	−1.9 ± 0.6	11.5 ± 2.6
**1.5M1.5**	52	83.8 ± 2.8	16.2 ± 1.89	94.4 ± 2.6	−1.1 ± 0.9	16.2 ± 1.9
**1.5M2**	21	85.8 ± 1.2	20.1 ± 1.19	92.7 ± 2.5	−0.81 ± 0.6	20.1 ± 1.2
**1.5M2.5**	13	86.1 ± 3.6	24.3 ± 2.52	90.1 ± 3.6	0.08 ± 1.7	24.3 ± 2.4
**2L2**	22	82.6 ± 1.8	20.3 ± 0.95	93.3 ± 2.0	−1.1 ± 0.5	20.3 ± 1.0
**2M1.5**	1	79.8	11.9	98.5	−1.8	11.7
**2M2.5**	1	81.8	24.7	92.3	−1.0	24.7
**2R2**	20	80.0 ± 1.2	20.4 ± 0.8	89.3 ± 1.4	0.23 ± 0.5	20.4 ± 0.8
**2.5L1**	7	78.3 ± 1.0	21.0 ± 1.9	89.5 ± 1.8	0.96 ± 0.5	21.3 ± 2.0
**2.5L1.5**	68	77.7 ± 2.6	17.9 ± 2.5	92.6 ± 2.0	−0.78 ± 0.5	17.9 ± 2.5
**2.5L2**	38	77.4 ± 2.6	21.1 ± 1.6	91.9 ± 1.6	−0.51 ± 0.7	21.1 ± 1.6
**2.5L2.5**	5	78.4 ± 1.6	24.0 ± 3.2	90.8 ± 0.8	−0.30 ± 0.3	23.9 ± 3.2
**2.5M1**	33	78.0 ± 2.2	14.3 ± 3.0	94.5 ± 4.1	−0.93 ± 0.8	14.2 ± 3.0
**2.5M1.5**	14	79.0 ± 1.7	15.8 ± 2.1	93.2 ± 3.8	−0.50 ± 1.1	15.8 ± 2.1
**2.5M2**	6	77.4 ± 2.6	21.1 ± 1.6	91.9 ± 1.6	−0.51 ± 0.7	21.1 ± 1.6
**2.5M2.5**	8	78.6 ± 2.3	27.0 ± 1.1	88.1 ± 0.8	0.90 ± 0.3	26.9 ± 1.1
**2.5M3**	5	78.5 ± 1.4	30.0 ± 1.5	88.2 ± 1.2	0.94 ± 0.6	30.0 ± 1.5
**2.5R1.5**	5	77.2 ± 2.3	18.5 ± 1.7	86.7 ± 2.8	1.10 ± 1.0	18.4 ± 1.6
**2.5R2**	7	78.4 ± 1.7	21.3 ± 2.1	87.5 ± 1.8	0.96 ± 0.7	21.3 ± 2.0
**2.5R2.5**	7	77.2±1.3	22.0±1.8	88.5±1.2	0.61±0.5	22.0±1.8
**3L2**	11	75.2 ± 2.7	23.5 ± 0.9	90.4 ± 1.1	−0.14 ± 0.4	23.5 ± 0.9
**3M1.5**	4	75.4 ± 2.3	20.0 ± 1.7	88.2 ± 0.1	0.63 ± 0.1	20.2 ± 2.1
**3M2.5**	10	74.6 ± 1.8	26.6 ± 1.1	88.2 ± 0.4	0.80 ± 0.2	26.6 ± 1.1
**3R2**	7	75.6 ± 1.2	22.8 ± 0.6	83.5 ± 2.9	2.59 ± 1.2	22.6 ± 0.5
**3.5L1.5**	35	71.2 ± 2.2	20.1 ± 1.8	90.1 ± 1.9	−0.01 ± 0.6	20.1 ± 1.8
**3.5L2**	18	72.6 ± 1.7	24.1 ± 2.1	89.9 ± 1.0	0.07 ± 0.4	24.1 ± 2.1
**3.5R2.5**	5	71.4 ± 2.0	28.7 ± 0.9	88.4 ± 0.9	0.84 ± 0.4	28.7 ± 0.9
**3.5M1**	50	70.6 ± 2.6	15.5 ± 2.1	91.1 ± 3.1	−0.21 ± 0.8	15.5 ± 2.1
**3.5M1.5**	11	72.8 ± 1.3	20.4 ± 1.7	87.9 ± 0.9	0.77 ± 0.3	20.4 ± 1.7
**3.5M2**	4	73.0 ± 0.7	23.8 ± 0.9	87.2 ± 0.2	1.20 ± 0.1	23.8 ± 0.9
**3.5M2.5**	20	72.6 ± 1.4	27.7 ± 3.0	86.7 ± 0.8	1.61 ± 0.5	27.6 ± 3.0
**3.5M3**	9	73.8 ± 5.6	35.5 ± 3.1	83.0 ± 2.6	4.28 ± 1.7	35.1 ± 3.1
**3.5R1.5**	8	71.3 ± 2.6	19.8 ± 0.9	85.1 ± 1.4	1.70 ± 0.6	19.8 ± 0.8
**3.5R2.5**	15	70.0 ± 2.3	24.4 ± 1.3	85.0 ± 1.5	2.15 ± 0.7	24.3 ± 1.3
**3.5R2.5**	16	70.8 ± 1.6	28.3 ± 2.2	84.7 ± 2.7	2.71 ± 1.5	28.2 ± 2.1
**4L2**	6	68.4 ± 1.4	26.6 ± 1.3	88.6 ± 1.1	0.63 ± 0.5	26.6 ± 1.3
**4M1.5**	8	68.9 ± 0.8	21.9 ± 1.1	86.4 ± 0.6	1.39 ± 0.2	21.8 ± 1.1
**4M2.5**	4	67.9 ± 0.9	30.1 ± 1.0	86.1 ± 0.7	2.08 ± 0.4	30.0 ± 0.5
**4R2**	4	68.4 ± 0.5	26.2 ± 1.3	82.5 ± 0.9	3.40 ± 0.3	26.0 ± 1.3
**4.5M1**	20	62.5 ± 2.5	17.7 ± 2.9	85.8 ± 5.3	1.36 ± 1.5	17.6 ± 2.9
**4.5M1.5**	43	64.9 ± 1.3	22.7 ± 2.4	86.5 ± 2.5	1.45 ± 1.0	22.6 ± 2.4
**4.5M2**	13	64.3 ± 2.1	26.8 ± 1.6	84.7 ± 2.6	2.50 ± 1.3	26.6 ± 1.6
**4.5M2.5**	24	65.2 ± 1.5	31.8 ± 2.6	82.7 ± 1.8	4.05 ± 1.2	31.5 ± 2.5
**4.5M3**	10	67.2 ± 2.2	41.0 ± 2.7	80.5 ± 1.7	6.68 ± 1.2	40.4 ± 2.7
**5M1.5**	9	59.0 ± 2.5	26.4 ± 1.4	83.8 ± 3.0	2.84 ± 1.3	26.2 ± 1.4
**5M2.5**	9	56.8 ± 3.4	35.8 ± 1.5	78.5 ± 2.0	7.16 ± 1.3	35.1 ± 1.5

**Table 2:** Difference in colour (ΔEab*) in the 49 “intermediate shades” of the 3D Master System.

		*n*	Percentage
Perceptibility colour difference	ΔEab* < 2.6 units	30	2.6%
Perceptible but clinically acceptable colour difference	2.6 < ΔEab* < 5.5 units	177	15.0%
Clinically unacceptable colour difference	ΔEab* > 5.5 units	969	82.4%
